# Gait Analysis of Hemiparetic Adult Patients with a Quadripod Cane and a Rolling Cane

**DOI:** 10.3390/healthcare12040464

**Published:** 2024-02-12

**Authors:** Bérengère Maillard, Mohamed Boutaayamou, Helena Cassol, Laurence Pirnay, Jean-François Kaux

**Affiliations:** 1 Physical Medicine, Rehabilitation and Sports Traumatology Department, University Hospital of Liège, 4000 Liège, Belgium; hcassol@chuliege.be (H.C.); jfkaux@uliege.be (J.-F.K.); 2 Department of Electrical Engineering and Computer Science, University of Liège, 4000 Liège, Belgium; mboutaayamou@uliege.be; 3 Laboratory of Movement Analysis (LAM-Motion Lab), University of Liège, 4000 Liège, Belgium

**Keywords:** gait analysis, hemiparesis, stroke, rolling cane, quadripod cane

## Abstract

Stroke consequences include hemiparesis and difficulty walking. Several types of canes exist to overcome these alterations, but little data compares the quadripod cane and the rolling cane in hemiparetic patients. The objective of this work is twofold: to determine whether the gait speed—the most often used parameter to assess gait performance—depends on the type of cane, and to establish which spatiotemporal parameters have the most influence. Thirty-four hemiparetic patients performed 10 m walking tests at comfortable and fast speed conditions, using both canes on two different days. To objectively analyze their gait patterns, we used a tri-axial Inertial Measurement Units (IMU)-based system to record the walking signals from which we extracted the gait spatiotemporal parameters. We particularly examined the speed, stride length, and durations of stance, swing, and double support phases. The results showed that hemiparetic patients walked faster with the rolling cane during both speed conditions. These speed increases could be explained by the decrease in the stance phase duration of the affected leg, the decrease in the double support duration, and the increase in cadence. Our findings suggest that the rolling cane allows safe and faster walking.

## 1. Introduction

A stroke is an acute onset of a central neurological lesion of vascular origin (ischemic or hemorrhagic) [1]. Stroke can be responsible for several alterations: cognitive, motor (weakness, loss of voluntary movement), sensitive, and proprioceptive (could affect balance control) [2].

Worldwide, stroke is the second most common cause of death after myocardial infarcts, and is the fourth most common cause of disability among adults over 65 years old [3,4,5].

In 2012, on a global scale, stroke caused 6.7 million deaths. The World Health Organization estimates that by 2030, it will be responsible for 13.7% of deaths [5]. The International Classification of Functioning Disability and Health describes health conditions (e.g., vision, hearing, walking, learning, and memory) and health-related domains (e.g., mobility, education, and social interactions in society) through a common language, including describing gait at different levels/subcategories (body functions/movement; activities, and participation/mobility) [6]. The chapter “gait pattern function” includes hemiplegic gait, limping, paraplegic gait, spastic gait, and stiff gait pattern [6].

Hemiparetic gait is characterized by step variability, low speed, decreased cadence, and high spatiotemporal asymmetry. In addition, the swing and phase durations of the affected limb are, respectively, longer and shorter than those of the healthy limb [1,7]. Callegri et al. conducted a gait analysis study of hemiparetic patients using electromyography [8]. Compared to the normal gait pattern, they observed early co-activation of the tibialis anterior and gastrocnemius muscles in the stance phase of hemiparetic patients [8]. The hemiparetic gait pattern is also characterized by a decrease in step length [1,7,9]. Low speed and step variability are correlated with an increased risk of falling, dependence, and co-morbidity, regardless of the post-stroke time [1,9,10,11].

In a prospective study including 800 patients after stroke, Jørgensen and collaborators have shown that at the end of the rehabilitation, 18% of patients were unable to walk, 11% walked with an aid (i.e., with the help of a therapist or using a technical aid), and 50% were able to walk independently (without an aid), while, unfortunately, mortality during rehabilitation was 21% [12].

Moreover, considering that 70% of patients experience a falls during the first year after a stroke, gait rehabilitation is therefore of paramount importance [3]. Patients find themselves in a vicious circle: they develop a fear of falling, so they stay at home and become socially isolated, which increases, in return, physical deconditioning and the risk of falling [3,9]. Walking, thus, could reduce comorbidities like, e.g., osteoporosis, cardiovascular disease, arthrosis, and obesity [13].

Thus, gait improvement is a priority since it provides greater autonomy and increases safety in daily activities. Furthermore, gait pattern in older patients is a marker of robustness and good health, and gait alteration is an evident sign of functional decline [14,15]. Early rehabilitation improves neuroplasticity and increases the chance of recovery [9].

To improve their rehabilitation and establish personalized programs, it is important to understand the gait pattern alteration by, e.g., measuring gait spatiotemporal parameters [7,14].

With hemiparesis, balance control is compromised in part by two elements. First, patients often have proprioceptive troubles [1,16]. These troubles change verticality perception and cause postural behaviors, such as pushing to their paretic side, which can hinder gait recovery [1,16]. The balance impairment is associated with an asymmetry of body weight repartition between both lower limbs [1]. Indeed, there is more load on the unaffected limb, which causes asymmetry of swing and stance time in the affected limb [1,16]. This could be related to the loss of capacity of the affected limb to support the body weight [1]. However, this phenomenon could be partially compensated for by using a cane to allow better balance control, which is an essential condition for independent walking [16].

To assess in each case whether it is convenient to use a cane, Guillebastre et al. have conducted a posturography analysis on the limb weight-bearing asymmetry (percentage of body weight on each limb) and instability (displacements of the center of pressure along the mediolateral axis) [16]. Their results showed that patients require a walking aid when they are unable to support at least 40% of their body weight on their paretic leg [16].

There are different types of walking aids, e.g., a cane, crutch, walker, and rollator. In order to choose the most appropriate aid, caregivers need to consider physical status, cognitive capacity, and patient preferences [9,16].

In the present study, we focused on two types of canes, the quadripod cane (Q) and the rolling cane (W). These canes have the advantage of remaining upright thanks to their four feet or four rolls, respectively, which can be comfortable for a population with balance disorders. In addition, they could be used in early rehabilitation stages.

The study [9], by Deltombe et al., is the only one we found on this subject. They showed that walking speed as an outcome of the 10MWT and 6MWT, and the total distance covered in the 6MWT, are greater when using the rolling cane compared to the quadripod cane. These two parameters can be improved without increasing the risk of falling [3]. The calculation of walking speed in [9] relied, however, on the use of a stopwatch, which cannot enable a refined understanding of the spatiotemporal parameters’ role in the increase in these walking speeds and total distances. Such refined gait analysis is lacking in the literature.

In this paper, we thoroughly analyze the gait patterns of hemiparetic patients using an ambulatory tri-axial Inertial Measurement Units (IMU)-based system, including developed hardware parts and associated signal processing algorithms. The objective is to investigate whether the rolling cane can help in improving gait speed when a patient walks at comfortable and fast speed conditions in a clinical context. The original contribution of this work involves quantifying with high accuracy and precision, for the first time, the spatiotemporal gait parameters that are the most decisive for the variation of the gait speed.

## 2. Materials and Methods

### 2.1. Design, Recruitment, Participants

This monocentric randomized crossover study was carried out in accordance with the recommendations of the ethics committee of the Faculty of Medicine of the University of Liège, Belgium, and was approved by it. All participants were volunteers, and they (or their legal representative) completed a written informed consent in accordance with the Declaration of Helsinki. The study registration number on ClinicalTrial.gov is NCT05163444.

The walking tests were performed on patients from two rehabilitation centers of the University Hospital of Liège: the “Centre Neurologique de Réadaptation Fonctionnelle de Fraiture” (CNRF) and the “Centre de réadaptation d’Esneux Ourthe-Amblève” (CHUOA).

We have recruited 34 hemiparetic patients from 15 February 2022 to 22 February 2023. Physiotherapists and doctors helped us in recruiting the participants. We informed them by email, posters, and discussions. Our sample size was calculated based on results obtained by Deltombe et al. [9]. We recruited patients who became hemiparetic after a stroke or after a traumatic brain injury if hemiparesis was the only motor deficit.

Inclusion and exclusion criteria are detailed in Table 1.

### 2.2. Materials

#### 2.2.1. Types of Canes

**Figure 1:**
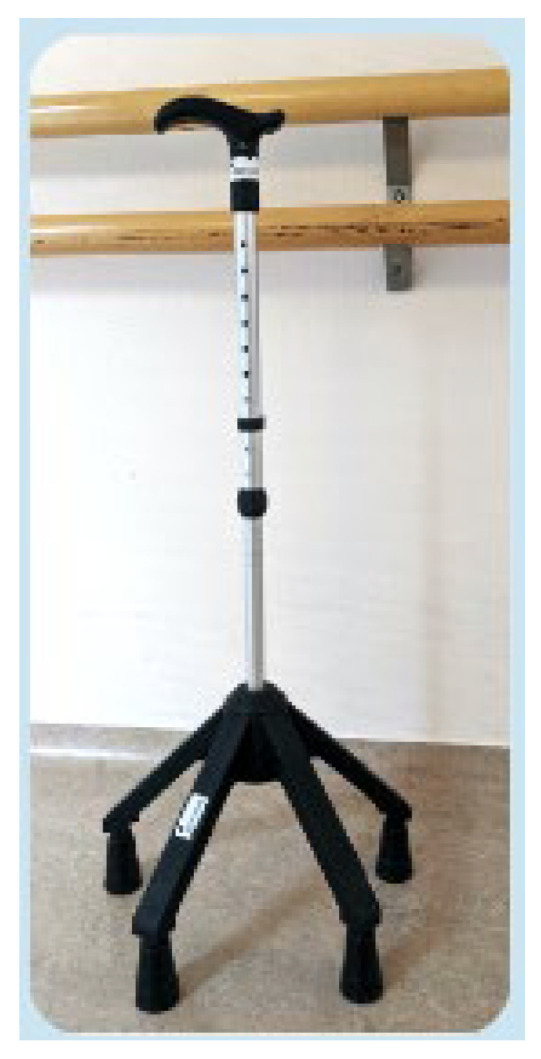
The quadripod cane.

Figure 1 shows the quadripod cane that was used. It includes four fixed and stable feet. Thanks to its stability, the quadripod cane is the only model of canes that allows full-body weight bearing. It is often used during neurological rehabilitation. It involves a 3-times gait because it requires a break during gait while the patient takes the cane off the floor.

**Figure 2:**
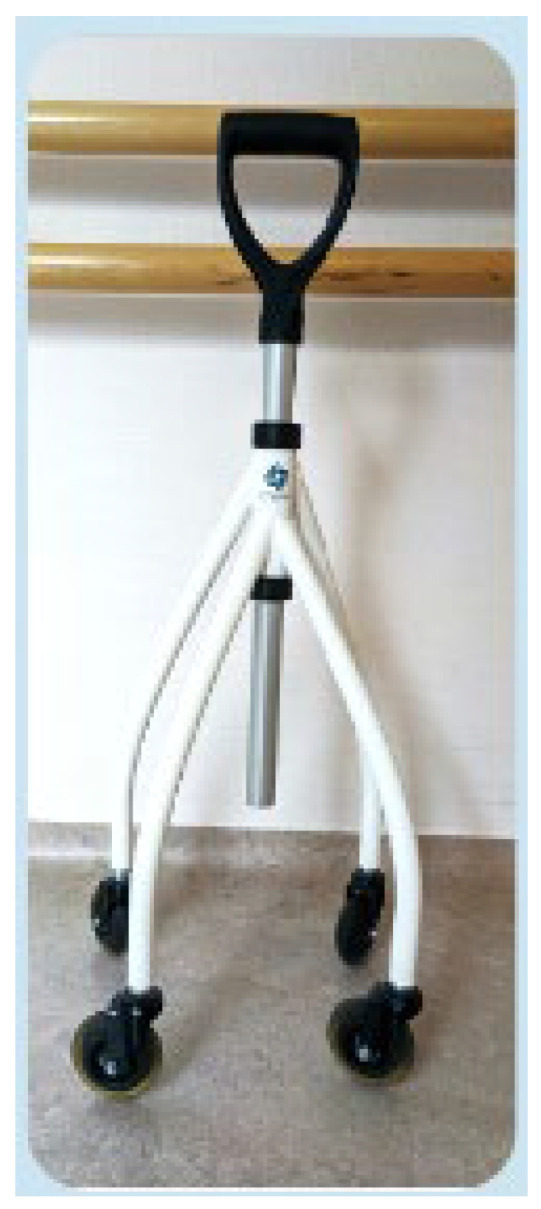
The rolling cane (InnoRehab, Wheeleo©).

Figure 2 shows the rolling cane that was used (Wheeleo©, InnoRehab, Ottignies, Belgium). It is equipped with four wheels. It allows for stable and easier movement with continuous support. The patient does not need to lift it, which enables a 2-times gait (i.e., the patient moves the cane while they are walking). This walking scheme may present the advantage of providing permanent support during each step of the gait.

#### 2.2.2. Gait Analysis System

We carried out this gait analysis using a wearable system that includes four IMU sensors connected by wires to a receiver module [10]. The latter has been developed at the Faculty of Applied Sciences (FAS) of the University of Liège. This innovative system combines (1) a portable hardware part that can robustly and continuously measure gait signals, and (2) validated signal-processing algorithms that can accurately and precisely quantify the spatiotemporal gait parameters based on these recorded signals.

This system was extensively validated by comparing its extracted spatiotemporal gait parameters to those concurrently provided by reference systems, including a kinematic analysis system and a force plate [10]. It has also been validated for slow-, comfortable-, and fast-speed conditions [17].

**Figure 3:**
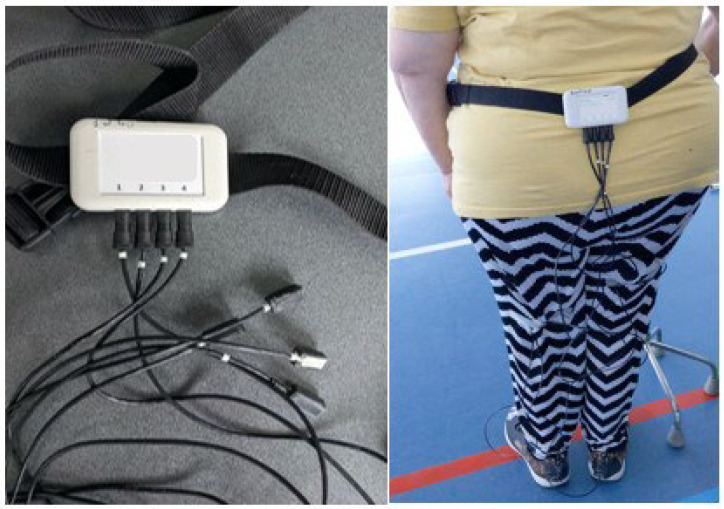
Tri-axial Inertial Measurement Units.

Each sensor consists of a three-axis accelerometer and a three-axis gyroscope. All the sensors have been integrated into very small packaging modules (2 cm × 0.7 cm × 0.5 cm) and are numbered from one to four [10]. Their attachment to the patient’s regular shoes has been standardized at the level of heels and toes to ensure robust data measurement. The accelerations and angular velocity signals are measured at the frequency of 200 Hz and stored in the receiver module (8.3 cm × 5.1 cm) (Figure 3) [10]. The frequency of 200 Hz has been used to ensure an accurate and precise extraction of the gait event timings from the acceleration data. For example, the initial contact event occurs rapidly and its (accurate and precise) extraction needs to be performed in refined time intervals, such as those with a time step of 5 ms. This ambulatory system is reliable, light, miniature, and can be used in a clinical environment [17].

We applied the aforementioned signal algorithms to the measured gait signals to extract the consecutive initial contacts (ICs) and final contacts (FCs) of the affected (a) and non-affected (na) sides during the walking of hemiparetic patients. We denote hereafter these gait events by aICs, naICs, aFCs, and naFCs, respectively. In healthy people, these gait events would correspond to the left and right heel strikes and left and right toe-offs. However, for hemiparetic patients, initial and terminal contacts of the foot are unstructured and do not match with heel strikes and toe-offs. In addition, these algorithms used these ICs and FCs to quantify locally (i.e., at the level of an individual gait cycle) several spatiotemporal parameters, such as the duration of the stance phase, swing phase, single stance, and double support, as well as the stride length, gait cycle speed, step variability, etc. [10]. As illustrated in Figure 2, the typical gait cycle of an affected side starts with aIC and ends with aFC. We emphasize that the IMU-based system has the advantage of recording gait signals in a synchronous manner from the affected and non-affected sides, thereby enabling the calculation of the symmetry expressed by each of these parameters.

The spatiotemporal parameters have been calculated within a gait cycle “i” and on a step-by-step basis as follows:

− Sr (the stride duration): the time between two ICs of the same foot, i.e., aSr(i) = aIC (i + 1) − aIC(i) and naSr(i) = naIC (i + 1) − naIC(i).
− Sa (the duration of the stance phase): the duration of loading on one limb, aSa(i) = aFC(i) − aIC(i) and naSa(i) = naFC(i) − naIC(i).
− Sw (the duration of the swing phase): the time between an FC and the next IC, aSw(i) = aIC(i + 1) − aFC(i) and naSw(i) = naIC(i + 1) − naFC(i).
− St (the step duration): the time between consecutive ICs of the affected and non-affected feet, aSt(i) = aIC (i) − naIC(i) and naSt(i) = naIC (i) − aIC(i).
− Cad (the cadence): the number of strides performed during one second, aCad(i) = 1/aSr(i) and naCad(i) = 1/naSr(i).
− DS (the duration of the double support phase): the time between consecutive ICs and IFs of the affected and non-affected feet, aDS(i) = aFC(i) − naIC(i) and naDS(i) = naFC (i + 1) − aIC(i).
− SL (the stride length): the distance covered between two consecutive ICs of the same foot. We used the method described in [17] to quantify the individual aSL and naSL values based on the recorded heel acceleration and angular velocity signal [17]. Briefly, this method robustly detects zero-velocity update regions in these signals. Adequate initial conditions are then applied in these regions to minimize the integration drifts during successive strap-down integrations carried out at the level of individual strides.
− GS (the gait speed): the speed calculated in each gait cycle i, aGS(i) = aSL(i)/aSr(i) and naGS(i) = naSL(i)/naSr(i).

### 2.3. Procedure

All patients performed tests with both canes, the first day with one cane and the next day with the other type of cane. The order of use of the two types of canes was randomized by means of online software (https://www.sealedenvelope.com/simple-randomiser/v1/lists, accessed on 1 February 2024).

Consequently, patients were randomly assigned to two groups: group A (day 1 with the rolling cane), and group B (starting with the quadripod cane).

For all patients, we completed a form to record sociodemographic and anthropometric data (e.g., age, sex, height, weight, paretic side, type/age of lesion).

Then, the IMU sensors were attached to patients’ shoes. Patients wore the same pair of shoes for both testing days. Subjects who wore an orthosis to correct stepping were asked to put it on.

In order to avoid any possible discomfort during the walking test, each wire was attached to the leg with an adhesive tape to avoid discomfort.

Subjects started tests without trials, but they had already used both canes with their physiotherapist.

To assess walking, we chose a walking test validated in a neurologic population: the 10 m walking test (10MWT) at comfortable and fast speeds. This short distance is interesting because it enables us to estimate the capacity of walking at home [18]. It is easy to perform and is validated as a good marker to assess endurance and functional ambulation. In addition, the 10MWT has been shown to not be operator-dependent and it has a good test–retest reliability [11,18].

Our cohort always started tests with the 10MWT at a comfortable, self-selected speed. For the second test, patients covered the same distance, and the instruction was to walk as fast as possible, in a safe condition. Patients could sit for a few minutes to rest before the last test, according to their needs.

With patient agreement, we made a video for each test to help in the data analysis. For example, if the signal showed a quirk, we check the associated video for the reason why this event had happened.

An evaluator walked near the patient to ensure her/his safety, but always behind the patient in order to not influence their walking by, e.g., imposing a pace.

### 2.4. Statistics

Data have been processed using the software “MATLAB 7.6.0” and with the expertise of an engineer from the FAS of ULiège.

These parameters (Sr, Sa, Sw, St, Cad, DS, SL, and GS) are considered in the comparison of the rolling cane (W) to the quadripod cane (Q) during the 10MWT at a comfortable walk (CW) and a fast walk (FW).

In this paper, we focused on the steady-state walking periods by excluding the first two and last two strides during the signal processing stage.

To compare the walking parameters in our two conditions (CW and FW), we used the student’s paired-sample *t*-test if both parameters (from the same subject) followed a Gaussian distribution, or the Wilcoxon paired-sample test if one or both parameters did not follow a Gaussian distribution.

Quantitative variables that followed a normal distribution were expressed using mean and standard deviation; otherwise, data were expressed as the median and interquartile range.

The results were considered significant when *p*-value < 0.05.

For each significant difference, we measured a performance index (Perf Index (%) = (Wvalue − Qvalue)/Qvalue). This index represents the difference between the two canes for the same parameter, in percentage.

We have calculated a ratio to make an evaluation of each phase duration proportion in relation to stride duration.

## 3. Results

The description of the population is detailed in Table 2. Patient with traumatic brain injury presented only a right hemiparesis.

We compared the parameters obtained when using the quadripod cane and the rolling cane for the affected limb. GS and SL were normalized by dividing their values by the patient’s height and the corresponding obtained parameters are denoted by GSn (s^−1^) and SLn (dimensionless), respectively.

First, during the comfortable walk, we observed several significant values, presented in Table 3.

With the rolling cane, GS has increased by 35%. Sr and St are, respectively, 22% and 17% longer with the quadripod cane in comparison with the rolling cane. Sa, that is, the time of single support on the paretic limb, is 27% shorter with the rolling cane. There is not a significant difference in Sw duration between both canes. We show an increase of 23% for the cadence with the rolling cane compared to the quadripod cane. DS is longer with the quadripod cane, with a difference of 31%.

Then, we analyzed the ratio of different parameters with respect to the stride duration. The DS ratio is the only ratio that shows a significant difference between both canes.

Table 4 provides the results obtained at the fast speed condition.

We observe the same parameter tendency as aforementioned during the CW condition. During the fast walking, the speed has been improved, using the rolling cane, by 38%.

Sw and SL are not significantly different between both canes. Sa is shorter with the rolling cane. The difference with the quadripod cane is 22%.

Sa difference between both canes is more pronounced at CW. Indeed, at CW, Sa is 27% longer with the quadripod cane, while it is 22% longer during FW.

The DS ratio is significantly longer with the quadripod cane (21.4%) compared to the rolling cane (17.2%).

## 4. Discussion

The primary goal of this work was to compare the gait speed of hemiparetic patients who walked using two types of canes. We have chosen the gait speed as a primary outcome due to its importance in assessing the gait performance [13].

Our secondary goal was to establish which spatial or temporal parameter has the most influence.

Some previous studies considered the use of an inertial sensor-based system with the hardware part located at the lower back. For example, Cho et al. used such a system to assess the effects of joint mobilization and active stretching on ankle joint range of motion and gait in stroke patients [19]. However, these systems cannot quantify the gait parameters on a step-by-step basis, and provide only global information (i.e., only the mean values of these parameters, e.g., [20]), which cannot reflect the evolving property of a pathological gait pattern.

Our innovative IMU-based system, on the other hand, can quantify several gait spatiotemporal parameters on a step-by-step basis, thereby enabling the calculation of parameters reflecting the gait variability. Indeed, in patients with Parkinson’s disease, for example, the mean values of spatiotemporal parameters are comparable to those found in healthy people, whereas there is a high step variability due to freezing and step length variability [20].

Another benefit of our system is that the gait analysis is now available in a clinical environment and usable in clinical practice [20]. It is indeed useful to avoid the travel of neurologic patients to a motion control lab. Moreover, we can use this system to multiply gait analysis to provide a longitudinal follow-up. In addition, it can help to assess therapeutic intervention efficiency (antispastic drug, effect of botulinum toxin, or effect of ankle–foot orthosis).

Here, we were only interested in the spatiotemporal parameters of the affected limb during the 10MWT at comfortable and fast speeds.

We showed that hemiparetic patients can walk significantly faster with a rolling cane whether it is at a comfortable or at a fast speed.

During the comfortable walk with the rolling cane (0.436 m/s), the speed is higher than the fast walk test with the quadripod cane (0.416 m/s), this shows how the rolling cane facilitates ambulation. Deltombe et al. have shown that these two values are the same (0.44 m/s), that is, gait speed at a comfortable walk with the rolling cane is equal to gait speed at a fast walk with the quadripod cane [9]. Both studies demonstrate that using the rolling cane improves gait speed.

Our results are consistent with those obtained by Deltombe et al., however, we find that speeds are slightly lower. In an attempt to explain this, we point out the following hypotheses. First, the mean age of our cohort is higher. Secondly, to measure gait speed, software systems helped to minimize stopwatch errors [18]. Finally, our last assumption is related to the calculation method. They have calculated the average speed at which patients covered 10 m, while in our study, we calculated the speed of the affected limb using the aforementioned step-by-step method, i.e., the speed corresponds to the average individual velocities of this limb.

To be more accurate, we calculated the normalized gait speed because this latter seems to be more influenced by height than by gender. Indeed, we examined the results of two studies [13,14]. They compared normalized gait speed according to gender, and they made the same observation for the two walk speeds (comfortable and fast): there is no significant difference between the sexes [13,14]. Thus, that indicates that gait speed rather depends more on height than on gender. We find the same observations, in fact, there is no significant difference when we calculate the normalized speed (Table 5).

The original contribution of this work involves quantifying the spatiotemporal gait parameters that have the most influence on speed improvement. Since the gait speed is given by SL/Sr, the results showed that the increase in speed using the rolling cane is explained by the decrease in the stride duration, since there is no significant difference in the stride length between both canes. In addition, the decrease in the stride duration is explained by the decrease in the stance duration, since the swing duration showed no significant difference between the two canes.

The gait difference between both canes could be then explained mainly by the decrease in the stance duration.

Regarding the paretic limb, during the CW, the gait speed is higher with the rolling cane (0.436 m/s) due to the decrease in the Sa duration (1.028 s). These values, for the quadripod cane, are, respectively, 0.324 m/s and 1.403 s.

Gillain et al. have studied spatiotemporal parameters of gait in healthy elderly people [14]. In alignment with their findings, we too observed an increase in gait speed while the stance phase duration decreased.

When we compare the ratio of the stance phase duration between the affected and the healthy limb, it is higher for the latter (Table 6). This suggests that instability on the affected limb is compensated by an increase in support on the healthy side, which has also been found in the study by Wang et al. [7].

If we check our data and compare Sw for the affected and the healthy limb, there is a significant difference, and that difference is apparent at both the comfortable and fast walk speeds. We found that Sw was always shorter in the healthy limb (regardless of the speed condition or the type of cane) (Table 6 and Table 7). The increasing duration of Sw for the paretic leg is due to impotence caused by stiffness and weakness [7]. This observation was reported in the study of Wang et al. [7]. In their study, walking tests were performed on a treadmill and they determined that patients who were usually walking with a cane could use a handrail. Swing duration asymmetry is mainly observed for patients using handrails because, for hemiparetic patients, stance phase duration is higher for the healthy side while swing duration is higher for the affected limb [7].

Furthermore, in healthy elderly people during CW, the DS ratio is 14% while this value is 23% with the quadripod cane and 20% with the rolling cane, which is significant [15]. Wang et al. also described an increase in DS duration for hemiparetic patients [7]. It could be explained because of trouble with balance control, especially during single support of both legs. Indeed, on the one hand, the paretic leg is too weak to support the body weight and, on the other hand, balance on the healthy limb is compromised due to alteration of verticality perception (pusher syndrome).

Otherwise, regarding gait speed, several studies have assessed that a functional gait speed is at least 0.80 m/s. Indeed, to cross the road (in accordance with traffic signal time), gait speed for daily life must be at least 0.80 m/s [1]. We find the same cut-off in a study by Perry and collaborators [21]. They established a classification according to patients’ walking ability after stroke [21]. In their cohort, they differentiate physiological walkers (house ambulation), for whom mean velocities were 6 m/min (0.1 m/s), and community walkers, who are walking at an average speed of 48 m/min (or 0.8 m/s) [21].

This gait pace is considerably lower than in healthy aging people but sufficient for functional ambulation in public places like commercial streets [21]. As a comparison, gait speed in aging healthy women is 1.26 m/s and 1.4 m/s in elderly men [14].

Although the walking speed in our study is lower than the threshold of 0.80 m/s (i.e., the community walking speed), hemiparetic patients walked faster with the rolling cane and approached an efficient speed. In this study, we recruited hemiparetic patients immediately after they started their rehabilitation. If they can adequately walk at the early stage of this rehabilitation, it is anticipated that they will increase their gait speed during the following months. Further instrumental sessions—using the present IMU-based system—could be carried out for the follow-up of these patients to assess their gait speed after, e.g., 6 or 12 months.

This study demonstrates that at two speeds (comfortable and fast), walked faster with the rolling cane. We explain this difference mainly by the reduction in the stance phase duration on a paretic limb. So, the rolling cane could be used more often in rehabilitation centers. We have collected lots of raw data for both paretic and healthy legs. We will then consider, in the next study, the analysis of the symmetry and variability outcomes.

Moreover, this work allows us to familiarize ourselves with an innovative gait analysis system. It could detect early frailty in elderly people (risk of falls) or neurological diseases in which gait impairment is a pre-clinical sign (e.g., Parkinson’s disease) to implement preventive measures [10,17,22].

On the other hand, IMU is a reliable tool to ensure a follow-up and check the efficiency of rehabilitation treatments (physiotherapy, correction by orthosis, or after toxin botulinic injection).

Regarding the limitations of our study, we should mention that we had some difficulties assessing all patients at the same time of the day. Indeed, for hospitalized patients, we depended on their rehabilitation program. In addition, in our cohort, clinical panels are widely different. We might be more vigilant about the functional level and classify patients by category according to their abilities. We should have considered some scores, such as the Modified Ashworth Scale (MAS), Functional Ambulation Categories (FAC), and initial NIHSS, and we should have classified patients according to the severity of the deficit using, e.g., the MRC score.

Otherwise, our sample is heterogeneous regarding etiology and side of the affected limb. However, considering that we had no particular hypothesis concerning the effect of the affected side or etiology on the use of both types of canes, we based our inclusion criteria on hemiparesis only. In addition, we would like to point out that hemiparesis was the only motor deficit in the patient who suffered from a traumatic brain injury.

Our results could be not completely representative of community ambulation for the following reasons: (i) the ground of the hospital is more regular than the outdoor ground, (ii) the patients reported that they were stressed because they felt they were being observed by their doctor, and (iii) the patients reported being anxious about their performance, and sometimes they reported a fear of falling.

In addition, we had not evaluated the risk of falling. It would be addressed if we reported the number of times the examiner had to stabilize the patient.

Moreover, we could have calculated the oxygen consumption to check whether the energy cost of walking is influenced by the type of cane.

## 5. Conclusions

This work considered the quantitative gait analysis of hemiparetic patients who are walking with a quadripod cane and a rolling cane. We have collected data with a tri-axial Inertial Measurement Unit System. Then, we studied spatiotemporal parameters during comfortable and fast walking with both types of cane. We found that the gait speed was higher with the use of a rolling cane during comfortable and fast walking. The speed improved by 35% at a comfortable speed and by 38% at a fast speed. Based on our results, these differences are related to (1) a decrease in the stance phase duration of the affected leg, (2) a decrease in the double support duration, and (3) an increase in the cadence. We show that an increase in gait speed is a marker of robustness and allows better balance control. Gait improvement should therefore be a goal in post-stroke rehabilitation. As Hornby et al. have shown, the most important factor in improving gait quality and speed is to increase stepping exercise during rehabilitation [23]. Finally, regardless of the type of cane, let’s get our patients moving!

Gait disorders cause frailty and dependency on daily activities and precipitate the onset of comorbidity. Thus, achieving gait autonomy, even with the use of a waking aid, is a priority in rehabilitation, and a gait pattern analysis helps medical staff to adapt treatment and personalize rehabilitation programs. In future work, we will quantify and analyze the gait symmetry outcomes in hemiparetic patients.

## Figures and Tables

**Table 1:** Inclusion and exclusion criteria.

Inclusion Criteria	Exclusion Criteria
Age > 18 years old	Understanding disorder
Hemiparesis post-stroke or after traumatic brain injury	Acute orthopedic failure
Able to walk with a quadripod cane or a rolling cane	Other neurological disease with gait trouble (Parkinson’s disease, severe polyneuropathy…)
Able to understand instructions	

**Table 2:** Population description.

Characteristics of Our Cohort (N = 34)	
Women	13
Median Age (Years) (min–max)	65 (19–91)
Mean Height (cm) ± SD	170.1 (±7.2)
Mean Weight (kg) ± SD	76.6 (±16.4)
Mean BMI (kg/m^2^) ± SD	26.3 (±4.5)
Median Time Since Stroke (Months) (IQR)	3 (2–8)
Right Paresis	18
Stroke	33
Traumatic Brain Injury	1

**Table 3:** Gait spatiotemporal parameters for Q vs. W for the affected limb, during the comfortable walk (CW). Significant values are highlighted in bold with asterisk.

CW	Q Mean or Median Val	W Mean or Median Val	*p* Value	Performance Index
Sr [s]	2.098 [1.671; 2.709]	1.636 [1.438; 2.052]	0.003 *	−0.22
Sa [s]	1.403 [1.083; 2.040]	1.028 [0.870; 1.395]	0.003 *	−0.27
Sw [s]	0.642 [0.488; 0.816]	0.644 [0.470; 0.763]	0.670	0
DS [s]	0.453 [0.338; 1.075]	0.314 [0.214; 0.478]	0.008 *	−0.31
St [s]	1.119 [0.932; 1.796]	0.927 [0.795; 1.184]	0.007 *	−0.17
SL (m)	0.678 (±0.195)	0.729 (±0.206)	0.310	0.08
SLn	0.399 (±0.114)	0.430 (±0.121)	0.300	0.08
GS (m/s)	0.324 (±0.132)	0.436 (±0.169)	0.004 *	0.35
GSn (s^−1^)	0.191 (±0.079)	0.257 (±0.100)	0.004 *	0.35
Cad (n°Sr/s)	0.481 (±0.152)	0.590 (±0.141)	0.004 *	0.23
**CW**	**Q Mean or Median Ratio**	**W Mean or Median Ratio**	***p* Value**	
Sa [%]	68.0 (±10.8)	63.9 (±8.7)	0.095	
Sw [%]	32.0 (±10.8)	36.3 (±8.8)	0.083	
DS [%]	23.0 [18.4; 40.7]	20.0 [15.7; 24.1]	0.042 *	
St [%]	60.4 (±10.3)	56.5 (±6.0)	0.070	

**Table 4:** Gait spatiotemporal parameters for Q vs. W for the affected limb during fast walk (FW). Significant values are highlighted in bold with asterisk.

FW	Q Mean or Median Val	W Mean or Median Val	*p* Value	Performance Index
Sr [s]	1.654 [1.494; 2.299]	1.415 [1.302; 1.875]	0.006 *	−0.14
Sa [s]	1.152 [0.903; 1.571]	0.898 [0.792; 1.112]	0.003 *	−0.22
Sw [s]	0.608 [0.460; 0.751]	0.580 [0.435; 0.754]	0.800	−0.05
DS [s]	0.333 [0.267; 0.689]	0.255 [0.197; 0.329]	0.002 *	−0.23
St [s]	0.895 [0.796; 1.560]	0.833 [0.690; 0.993]	0.043 *	−0.07
SL (m)	0.741 (±0.232)	0.842 (±0.245)	0.094	0.14
SLn	0.416 [0.364; 0.550]	0.508 [0.396; 0.613]	0.080	0.14
GS (m/s)	0.416 (±0.171)	0.572 (±0.228)	0.003 *	0.38
GSn (s^−1^)	0.245 (±0.101)	0.338 (±0.134)	0.003 *	0.38
Cad (n°Sr/s)	0.557 (±0.158)	0.669 (±0.155)	0.006 *	0.2
**FW**	**Q Mean or Median Ratio**	**W Mean or Median Ratio**	***p* Value**	
Sa [%]	65.8 (±10.4)	61.4 (±8.1)	0.070	
Sw [%]	34.3 (±10.5)	38.7 (±8.2)	0.065	
DS [%]	21.4 [16.6; 29.2]	17.2 [13.4; 20.9]	0.012 *	
St [%]	59.1 (±9.9)	56.2 (±5.7)	0.15	

**Table 5:** Gait spatiotemporal parameters for Q and W: women vs. men during comfortable walk (CW). Significant values are highlighted in bold with asterisk.

		Women	Men	*p* Value
		Mean/Median	Mean/Median	
Quadripod Cane	SL [m]	0.62 (±0.16)	0.72 (±0.21)	0.140
	GS [m/s]	0.24 [0.20; 0.28]	0.35 [0.27; 0.46]	0.035 *
	SLn [-]	0.37 (±0.10)	0.42 (±0.12)	0.300
	GSn [1/s]	0.15 [0.12; 0.18]	0.21 [0.15; 0.26]	0.084
Rolling Cane	SL [m]	0.62 (±0.18)	0.804 (±0.19)	0.010 *
	GS [m/s]	0.38 (±0.16)	0.473 (±0.17)	0.130
	SLn [-]	0.38 (±0.12)	0.465 (±0.11)	0.042 *
	Gsn [1/s]	0.23 (±0.10)	0.274 (±0.10)	0.260

**Table 6:** Gait spatiotemporal parameters for Q: affected limb (A) vs. unaffected limb (nA) during comfortable walk (CW). Significant values are highlighted in bold with asterisk.

	A Mean/Median Values	nA Mean/Median Values	*p* Value
Sr [s]	2.098 [1.671; 2.709]	2.061 [1.689; 2.728]	0.910
Sa [s]	1.403 [1.083; 2.040]	1.705 [1.299; 2.287]	0.097
Sw [s]	0.642 [0.488; 0.816]	0.400 [0.328; 0.440]	0.000 *
DS [s]	0.453 [0.338; 1.075]	0.424 [0.286; 0.610]	0.310
St [s]	1.119 [0.932; 1.796]	0.829 [0.688; 0.995]	0.000 *
SL (m)	0.678 (±0.195)	0.811 (±0.212)	0.011 *
GS (m/s)	0.285 [0.233; 0.419]	0.364 [0.287; 0.418]	0.077
SLn	0.399 (±0.114)	0.476 (±0.117)	0.010 *
GSn (s^−1^)	0.171 [0.134; 0.246]	0.215 [0.169; 0.242]	0.090
Cad (n° of Sr/s)	0.481 (±0.152)	0.478 (±0.152)	0.093
	**A Ratio**	**nA Ratio**	
Sa [%]	68.0 (±10.8)	80.8 (±6.6)	0.000 *
Sw [%]	32.0 (±10.8)	19.3 (±6.6)	0.000 *
DS [%]	23.0 [18.4; 40.7]	19.3 [15.2; 24.4]	0.049 *
St [%]	60.4 (±103)	39.7 (±10.4)	0.000 *

**Table 7:** Gait spatiotemporal parameters for W: affected limb (A) vs. unaffected limb (nA) during fast walk (FW). Significant values are highlighted in bold with asterisk.

	A Mean/Median Values	nA Mean/Median Values	*p* Value
Sr [s]	1.636 [1.438; 2.052]	1.632 [1.429; 2.043]	0.980
Sa [s]	1.028 [0.870; 1.395]	1.244 [1.039; 1.626]	0.017 *
Sw [s]	0.650 (±0.230)	0.401 (±0.080)	0.000 *
DS [s]	0.314 [0.214; 0.478]	0.319 [0.234; 0.507]	0.810
St [s]	0.927 [0.795; 1.184]	0.729 [0.628; 0.897]	0.001 *
SL (m)	0.729 (±0.206)	0.844 (±0.186)	0.022 *
GS (m/s)	1.636 [1.438; 2.052]	1.632 [1.429; 2.043]	0.980
SLn	0.430 (±0.121)	0.497 (±0.108)	0.022 *
GS (s^−1^)	0.257 (±0.100)	0.291 (±0.085)	0.160
Cad (n° of Sr/s)	0.590 (±0.141)	0.589 (±0.141)	0.980
	**A Ratio**	**nA Ratio**	
Sa [%]	63.9 (±8.7)	76.6 (±6.4)	0.000 *
Sw [%]	36.3 (±8.8)	23.5 (±6.5)	0.000 *
DS [%]	20.0 [15.7; 24.1]	19.2 [15.0; 24.8]	0.920
St [%]	56.5 (±6.0)	43.7 (±6.0)	0.000 *

## Data Availability

Data are available under request to the corresponding author.
